# Combining multi-scale 3D printing technologies to engineer reinforced hydrogel-ceramic interfaces

**DOI:** 10.1088/1758-5090/ab69d9

**Published:** 2020-02-19

**Authors:** Paweena Diloksumpan, Myléne de Ruijter, Miguel Castilho, Uwe Gbureck, Tina Vermonden, P René van Weeren, Jos Malda, Riccardo Levato

**Affiliations:** 1 Department of Clinical Sciences, Faculty of Veterinary Medicine, Utrecht University, The Netherlands; 2 Department of Orthopaedics, University Medical Center Utrecht, Utrecht University, Utrecht, The Netherlands; 3 Regenerative Medicine Center, Utrecht, Utrecht University, Utrecht, The Netherlands; 4 Department of Biomedical Engineering, Faculty of Engineering, Technical University of Eindhoven, The Netherlands; 5 Department for Functional Materials in Medicine and Dentistry, University Hospital of Würzburg, Würzburg, Germany; 6 Department of Pharmaceutics, Utrecht Institute for Pharmaceutical Sciences (UIPS), Faculty of Science, Utrecht University,The Netherlands; 7 Author to whomany correspondence should be addressed

**Keywords:** biofabrication, melt electrowriting, bioinspired interface, bone and cartilage tissue engineering, microfibres, ceramics

## Abstract

Multi-material 3D printing technologies that resolve features at different lengths down to the microscale open new avenues for regenerative medicine, particularly in the engineering of tissue interfaces. Herein, extrusion printing of a bone-biomimetic ceramic ink and melt electrowriting (MEW) of spatially organized polymeric microfibres are integrated for the biofabrication of an osteochondral plug, with a mechanically reinforced bone-to-cartilage interface. A printable physiological temperature-setting bioceramic, based on *α*-tricalcium phosphate, nanohydroxyapatite and a custom-synthesized biodegradable and crosslinkable poloxamer, was developed as bone support. The mild setting reaction of the bone ink enabled us to print directly within melt electrowritten polycaprolactone meshes, preserving their micro-architecture. Ceramic-integrated MEW meshes protruded into the cartilage region of the composite plug, and were embedded with mechanically soft gelatin-based hydrogels, laden with articular cartilage chondroprogenitor cells. Such interlocking design enhanced the hydrogel-to-ceramic adhesion strength >6.5-fold, compared with non-interlocking fibre architectures, enabling structural stability during handling and surgical implantation in osteochondral defects *ex vivo*. Furthermore, the MEW meshes endowed the chondral compartment with compressive properties approaching those of native cartilage (20-fold reinforcement versus pristine hydrogel). The osteal and chondral compartment supported osteogenesis and cartilage matrix deposition *in vitro*, and the neo-synthesized cartilage matrix further contributed to the mechanical reinforcement at the ceramic-hydrogel interface. This multi-material, multi-scale 3D printing approach provides a promising strategy for engineering advanced composite constructs for the regeneration of musculoskeletal and connective tissue interfaces.

## 1 Introduction

Establishing a secure integration between mechanically dissimilar materials is a major challenge in engineering interfaces between biological tissues. In musculoskeletal tissues, hard, mineralized materials are naturally integrated with soft tissues, for example, the cartilage-to-bone boundary. This interface plays a pivotal role in the regulation of intercellular communication, through the diffusion of bioactive molecules between the articular surface and the subchondral bone [1]. Such filter function, together with the transmission of mechanical loads [2, 3], contributes to maintaining homeostasis and hence functionality of the articulating joint. Traumatic injuries to the articular cartilage and degenerative processes can lead to osteoarthritis, which is a prevalent and debilitating condition in our aging population. This disorder may result in the disruption of the integrity of the subchondral bone, cartilage and their interface, urging the development of approaches that can functionally restore the affected tissue. Thus far, principally, the use of soft materials has been investigated for cartilage restoration, in particular those based on biocompatible hydrogels that can provide a highly hydrated environment aiding the encapsulation and differentiation of cells. However, these materials are difficult to integrate with stiff materials that constitute successful supports as regenerative scaffolds or prosthetic replacements for mineralized tissues, such as bone [4]. Nevertheless, specific classes of double-network hydrogel formulations have been designed to feature outstanding toughness and adhesion strength to ceramics and metals [5]. However, these gels are very stiff or composed of dense polymer networks that have not been proven as suitable to support homogenous neo-tissue matrix deposition from encapsulated cells [6, 7]. Several strategies for integrating soft hydrogels with stiff bone substitute have been developed [4, 8–11], including binding with adhesive glues [12], coupling through covalent chemical bonds [13], or forming compositional gradients using the same based material via casting [14]. A major drawback of these strategies is that the majority offer little to no control over the architecture of the engineered interface.

The recent advances in 3D printing and biofabrication technologies open new avenues for the creation of multi-material architectures that can mimic or replace biological interfaces. Medical imaging, such as computed tomography, can be used as blueprints to replicate anatomical features of the native osteochondral boundary [15]. The layer-by-layer fabrication approach, typical of additive manufacturing techniques, enables us to freely design different pore geometries across the depth of the bone and cartilage compartments [16], as well as to introduce gradients of bioactive cues and inorganic particles [17–22]. Additionally, even low-viscosity hydrogels with low ability to retain their shape post-printing have been precisely deposited into biphasic structures reminiscent of osteochondral units, for instance with the aid of sacrificial supporting baths [23], extending the array of cellfriendly materials usable in bone and cartilage bioprinting. Importantly, cell-laden hydrogels can be mechanically reinforced when printed in coordination with thermoplastic polymers [24–28] and even ceramics that set under cell-friendly conditions [29].

However, such co-printing methods result in the shielding of the soft hydrogels from mechanical loads and do not necessarily improve their binding ability to an osteal anchor. Moreover, most of these methods suffer from limited spatial resolution (typically ∼100 *μ*m) and thus cannot mimic micro- and sub-micron- scale features of the osteochondral interfaces.

A new solution for the generation of fully biofabricated osteochondral boundaries can come from combining printing technologies able to resolve details at different length-scales [30]. MEW has recently emerged as a high-resolution 3D printing method to create highly-ordered, thermoplastic microfibre meshes [31] in the micron and sub-micron range [32], allowing for multimodal scaffold fabrication [33]. These MEW meshes, when infused with cell-friendly hydrogels, create composite materials with improved shear properties and outstanding compressive properties approaching those of native cartilage [34]. Despite this potential, the development of material-based strategies to create bioinspired, reinforced interfaces using such microfibre deposition methods has not been reported yet.

Biomaterials like *α*-tricalcium phosphate (*α*-TCP) have been used as injectable bone regenerative materials due to their biocompatibility and osteoconductivity [35, 36]. The self-setting capacity of *α*-TCP through hydrolysis also results in products that have a structure comparable with the inorganic components of native bone [37]. These properties allow us to process *α*-TCP for making customized scaffolds, for instance as recently shown for developing printable bone cements [38]. However, there is a limitation to using *α*-TCP due to its high solubility which leads to fast degradation. Incorporation of other inorganic phases, for instance, nanohydroxyapatite, FDA approved in several biomedical products [39, 40], has been well-described to improve the osteogenic potential of the ceramic, both in terms of osteoinduction and osteoconduction [41]. Given these promising biological properties and the low-temperature setting reactivity, this system offers a unique opportunity for direct printing with low melting polymers, as explored in this work.

In this present study, we introduce a novel approach that combines different 3D printing technologies, with the aim to directly form a secure inte-gration at the interface between two mechanically distinct materials, particularly between cell-laden hydrogels and biologically relevant ceramics and polymers. To achieve that, a bioceramic ink that sets at physiological conditions, was developed based on a calcium phosphate (CaP) formulation that mimics the mineral phase of bone, and shaped as subchondral bone substitute using a pneumatic-driven extrusion-based printer. Next, microfibrous polymeric meshes obtained by MEW were directly anchored into the ceramic ink, and were embedded in a cell-laden soft hydrogel based on methacryloyl-modified gelatin (GelMA), to represent the cartilage component. Several microfibre structures were studied in their capacity as the interlocking agent to enhance the interfacial adhesion of the hydrogelceramic interface and as mechanical reinforcement to enhance the compressive properties of the hydrogel. This technology has been used to create fully biofabricated osteochondral plugs for the treatment of bone and cartilage defects.

## 2 Materials and methods

### 2.1 Preparation of the calcium phosphate-based paste

**Figure 1:**
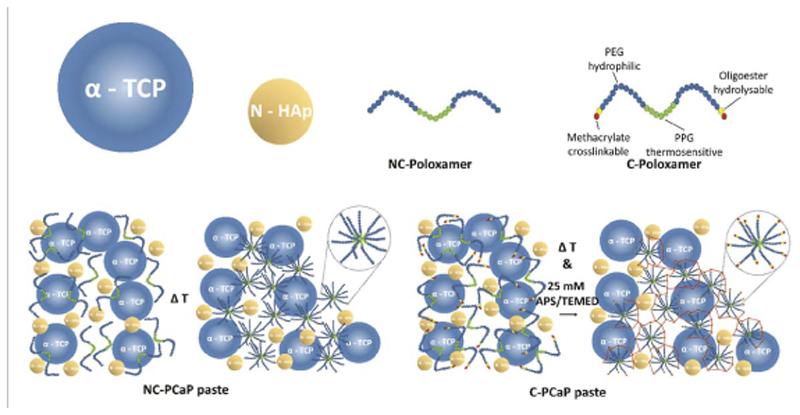
Material composition schematic pictures representing the compositions of the PCaP pastes.

The printable calcium phosphate (PCaP)-based ink, consisting of a particle and a liquid phase, was prepared in-house (figure 1). For 1 g of printable phase, 660 mg of milled alpha-tricalcium phosphate microparticles (*α*-TCP, average size 3.83 *μ*m, Cambioceramics, Leiden, the Netherlands) were mixed with 40 mg of nanohydroxyapatite (nano-HA, particle size <200 nm, Ca_5_(OH)(PO_4_)_3_, Sigma Aldrich). The liquid phase was composed of a 40% w/v hydrogel precursor solution, consisting of either unmodified poloxamer (Pluronic^®^ F-127, Sigma-Aldrich) or a custom-synthesized hydrolysable, crosslinkable poloxamer, whose terminal hydroxyl groups were modified by grafting caprolactone oligomers and methacryloyl groups (P-CL-MA, with 1 repeating unit for CL), as reported previously [42]. The unmodified (non-crosslinkable) and modified (crosslinkable) poloxamer were dissolved in PBS and PBS supplemented with 25 mM ammonium persulphate (APS, Sigma Aldrich), respectively. Prior to mixing, the particle and the liquid phases were stored at 4 °C for 30 min in order to prevent thermal gelation of the poloxamer component. Subsequently, either the non-crosslinkable (NC) or crosslinkable (C) poloxamer was added to particles and mixed manually by stirring for 3 min at 4 °C to ensure homogenous distribution of the particles. Subsequently, the prepared non-crosslinkable PCaP inks (NC-PCaP) and crosslinkable PCaP inks (C-PCaP) were loaded into a dispensing cartridge, closed with a retainer cap and stored at 4 °C until used.

### 2.2 Rheological characterization

Rheological characterization was performed on NC-PCaP, C-PCaP, NC-Poloxamer, C-Poloxamer and water-PCaP using a rheometer (Discovery Hybrid Rheometer (HR-2), TA instrument). The test was conducted on a Peltier plate with pre-set temperature of 20 °C. The test geometry was a 20mm diameter parallel plate. All measurements were performed while covering each sample with a solvent trap to prevent water evaporation from the composite material. The geometry gap was set to 300 *μ*m. Reactivity of the PCaP-based inks was assessed under oscillatory measurements at a frequency of 0.1 rad·s-1 and 0.1% strain, which is within the linear viscoelastic range (LVR) for all samples (supplementary figure S1 is available online at stacks.iop.org/BF/12/025014/ mmedia). Shear recovery measurements were carried out under oscillatory conditions by applying low and high strain cyclically. A low strain of 0.05% was applied for 300 s and then increased to 150% (outside LVR) for 300 s at the same frequency of 0.1 rad·s-1. These steps were repeated three times. Finally, steady-state flow measurements were performed in order to assess flow behaviour of the materials while applying shear rates from 0.001–1000 S-1. Consistency was ensured by repeating all measurements three times.

### 2.3 Printing of the bioceramic scaffolds

Bioceramic scaffolds were fabricated with pneumatic-driven, extrusion-based 3D (bio)printing equipment (3DDiscovery, regenHU, Villaz-St-Pierre, Switzerland). To optimize printing parameters, two layers of meander infill in a circle was designed as a printing path and eventually generated g-code by using Bio-CAD software (regenHU, Villaz-St-Pierre, Switzerland). The effects of extrusion pressure, translational speed of the collector plate and layer height on the diameter of printed strand were investigated, in order to optimize the printing resolution. The NC-PCaP ink was utilized initially for testing by extruding through a conical nozzle (inner diameter: 250 *μ*m) at ambient temperature (while maintaining temperature between 20 and 25 °C). The average diameter of printed strands from each printing setting was measured from stereomicroscopy pictures by using ImageJ software [43].

All printing settings for obtaining cylindrical filaments with precise alignment were selected. Additionally, the maximum designed strand-to-strand distance at which overhang filaments would retain their straightness without sagging to the lower layer was investigated. Based on the information, optimised porous cylindrical structures consisting of meandered infills in each layer were designed. After the printing process, the printed paste was allowed to set into a cement scaffold, through the hydrolytic conversion of the *α*-TCP microparticles into calcium deficient hydroxyapatite (CDHA) [44], via incubation in a saturated humidity environment at 37 °C for at least three days. Similar printing parameters and post-printing treatment was applied for the C-PCaP ink. Subsequently, C-PCaP scaffolds were immersed in 25mM tetramethylethylenediamine (TEMED, Invitrogen) solution at 37 °C for one hour in order to polymerize the crosslinkable poloxamer. Finally, C-PCaP structures were rinsed in PBS twice, and air dried at ambient temperature. When required for cell culture, the scaffolds were disinfected by immersion in 70% v/v ethanol, followed by exposure to UV light for 2 h.

### 2.4 Macroporosity of PCaP scaffolds

Porous cylindrical PCaP scaffolds (diameter: 5.0 mm, height: 5.0mm) were produced from either NC-PCaP paste or C-PCaP paste. Scaffolds were obtained by stacking meander pattern layers in a double alternated pattern (0°-0°-90°-90°), to ensure a consistent lateral pore size of 500 *μ*m. By varying the designed strand-to-strand distance from 600 to 800 *μ*m, NC and C scaffolds with four different macroporosity ranges were prepared: 20%–30%, 30%–40%, 40%–50%, and 50%–60% (N = 3–17). Porosity of printed PCaP scaffolds was determined by gravimetry analysis (equation (1)) [45].

(1) $$Totalporosity=1-\left(\frac{\rho_{Scaffold}}{\rho_{material}}\right).$$

Relative density of the used material (*ρ*_material_) was quantified as reported previously [46]. Actual dry weight of dense scaffolds, regardless of micro-porosity, was measured using mass scales. Average diameter and height of the scaffolds were measured by using digital Vernier callipers. Relative density of fabricated scaffolds (*ρ*_scaffold_) was calculated from actual dry weight and volume of porous scaffolds.

### 2.5 Mechanical characterization of the bioceramic scaffolds

Unconfined uniaxial compression tests were conducted on scaffolds with different ranges of macroporosity (20%–30%, 30%–40%, 40%–50%, and 50%–60% (N = 3–17)), using a system (MTS Criterion^®^ Electromechanical universal Test Systems, Model 42) equipped with a 500 N load cell. Samples were measured after equilibration in PBS for at least 30 min and subjected to a displacement ramp (0.5mm min^−1^) until failure. Raw data was used to calculate the compressive tangent modulus by measuring the slope of the linear region found in the range 0%-5% strain in the stress-strain curve, as well as ultimate strength and energy to failure using Matlab (R2018, MathWorks^®^).

### 2.6 Cell isolation and culture

Primary cells were obtained from healthy tissues (bone marrow and articular cartilage) of a deceased, skeletally mature equine donor (aged 6 years old; n = 1), donated for research by their owner, according to the guidelines of the Institutional Animal Ethical Committee of the veterinary clinic of Utrecht University. Mesenchymal stromal cells (MSCs) were harvested from bone marrow aspirated from the sternum, while articular cartilage-derived chondroprogenitor cells (ACPCs) were obtained from enzymatic digests of cartilage from the metacarpophalangeal joint, following previously reported protocols and following the ethical regulations of the host institution [47]. MSCs were expanded in minimum essential medium alpha (*α*MEM, 22561 Gibco, The Netherlands) supplemented with 0.2 mM L-ascorbic acid 2-phosphate (ASAP, Sigma), 10% fetal calf serum (FCS, Lonza, The Netherlands), 100 U/ml penicillin with 100 mg ml^−1^ streptomycin (Life Technologies, The Netherlands) and 1 ng ml^−1^ basic fibroblast growth factor (bFGF, Peprotech, UK). ACPCs were expanded in Dulbecco’s modified Eagle medium (DMEM, 31966, Gibco, The Netherlands), supplemented with 10% v/v FCS, 0.2 mM L-ascorbic acid-2-phosphate, 100 U/ml penicillin, 100 mg ml^−1^ streptomycin and 5 ng ml^−1^ (bFGF, Peprotech, UK)). Cells were used between passage 3 and 5.

### 2.7 *In vitro* cytocompatibility and osteogenic potential

The indirect cytotoxicity of the bioceramic ink was determined to evaluate the potential release of harmful compounds from the CDHA and from the hydrogel component of the cement scaffolds. Four formulations of PCaP were prepared by mixing the particle phase with different liquid compositions: distilled water, NC-poloxamer, C-poloxamer and 10% gelatin-methacryloyl (GelMA). GelMA synthesis was performed as previously reported [48]. Cast PCaP discs (diameter: 5.0 mm., height: 2.0 mm) were incubated in MSC expansion medium for 48 h before using. MSCs (10^4^ cells/well) were seeded on tissue-culture treated poly-styrene and cultured with eluates of the PCaP scaffolds. The PCaP-exposed medium was exchanged every two days. Cells exposed to MSCs expansion medium supplemented with 0.1% v/v Tween-20 were used as negative control. Cell metabolic activity was assessed with a resazurin assay (AlamarBlue™ Cell Viability, Invitrogen). Next, proliferation and osteogenic differentiation of cells that were in direct contact with the PCaP scaffold were assessed. To enhance the number of seeded cells on the scaffold, porous cylindrical C-PCaP scaffolds (diameter: 13.0 mm, height: 1.0mm) were printed with single alternated pattern (0°-30°-60°-90°) and a designed strand-to-strand distance of 750 *μ*m.

Firstly, MSCs were seeded onto the scaffolds (5 · 10^4^ cells/scaffold, n = 4 per time point) and cultured in the expansion medium, supplemented with 10 mM N-2-hydroxyethylpiperazine-N-2-ethane sulfonic acid (HEPES, Gibco) to assess cell proliferation. At day 1, 3, 7 and 14 the cell-laden scaffolds were collected and cell lysates were obtained by the addition of the protein extraction buffer M-PER (Thermo Scientific). The amount of cells at each time point was quantified by measuring lactate dehydrogenase activity in the lysate (LDH-kit, Roche diagnostic GmBH). Additionally, cell-laden scaffolds at each time point were washed in PBS, fixed with phosphate buffered formalin (pH 7.2), and stained for actin with phalloidin conjugates FTIC (Sigma) for 30min to observe cell morphology. Nuclei were counterstained with 4′,6-diamidino-2-phenylindole (DAPI, 100 ng ml^−1^, Sigma) for 1 min. Secondly, MSCs were seeded on bioceramic constructs (10^5^ cells/ scaffold, n = 4 per analysis) and cultured in the expansion medium, supplemented with 20mM β-glycerol phosphate, 100 nM dexamethasone and 10mM HEPES to assess osteogenic differentiation. The medium was refreshed every two days. At day 1, 7, 14, and 21 cell-laden scaffolds were lysated in M-PER and alkaline phosphate (ALP) activity was measured performing a p-nitrophenyl phosphate assay (SIGMAFAST™, Sigma Aldrich), together with DNA content, determined using the Quan-iT-Picogreen-dsDNA kit (Molecular Probes, Invitrogen, Carlsbad, USA). Formalin-fixed constructs were also labelled with DAPI and immunostained for the osteoblastic marker osteonectin (primary antibody, secondary antibody, Alexafluor 546 (goat anti-mouse, 1752107 Life technologies)). Fluorescently stained constructs were imaged with a confocal laser scanning microscope (TCS SP8, Leica, Netherlands).

### 2.8 Fabrication of multiphasic hydrogel-thermoplastic-bioceramic composite scaffolds mimicking an osteochondral plug

**Figure 2:**
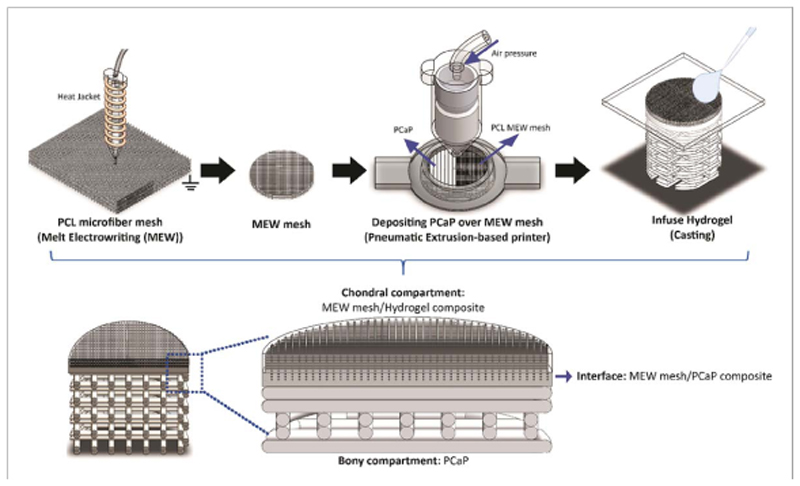
Fabrication process of the osteochondral construct by using a combination of different 3D printing techniques.

Polycaprolactone (PCL) microfibre meshes were fabricated from medical-grade polycaprolactone (Purasorb^®^ PC 12 Corbion PURAC, The Netherlands) using a custom-made melt electrowriting device as previously described [49]. MEW printing parameters were: printing temperature of 90 °C, collector velocity of 50mm s^−1^, voltage of 10 kV, and pressure of 1.5 bar. Printing was performed at room temperature (22–24 °C) with a humidity between 30%–50%. By using these settings, microfibre meshes organized in orthogonal square box patterns (fibre diameter = 10 *μ*m, fibre spacing = 300 *μ*m, total height = 1.3mm) were obtained, which were later cored to obtain 8mm diameter cylinders using a biopsy punch. These cylindrical meshes were then fixed on a glass slide using a custom-made holder and placed onto the collecting platform of the extrusion-based printer. C-PCaP paste was directly printed over the MEW-printed microfibre mesh, to form a 6.3-mm diameter bioceramic scaffold. The initial height for depositing the C-PCaP paste was optimized thoroughly to ensure printing without damaging the architecture of the PCL microfibres. The first two layers were generated without macro-porosity to mimic the subchondral bone plate. The following layers were deposited with a designed strand-to-strand distance of 700 *μ*m, forming a bone-mimetic osteal anchor. After letting the ceramic component set at 37 °C, the MEW mesh was infused with a 10% w/v GelMA solution [50] in PBS, supplemented with 25mM APS/ TEMED to allow chemical crosslinking of the hydrogel, thus completing the cartilage mimetic-region of the engineered osteochondral plug (figure 2). Finally, the overall construct was removed from the mold and transferred into 25 mM APS/TEMED supplemented PBS at 37 °C for one hour to allow completion of crosslinking of both the C-poloxamer in C-PCaP and GelMA hydrogel.

### 2.9 Interfacial hydrogel-ceramic adhesion strength

The strength of the interconnection at the interface between microfibre-reinforced hydrogel and the bio-ceramic scaffold was determined using a Dynamic Mechanical Analyzer (DMA Q800, TA Instrument), modified with ring-shaped custom-made sample holders. Additively manufactured samples were mounted so that the C-PCaP and hydrogel compartments were lodged each into the circular cavity of a holder. These holders were then displaced in the direction parallel to the ceramic-hydrogel interface applying a force ramp, until the two parts were completely separated. Shear stress and energy at failure were calculated respectively. Experimental groups consisted of GelMA: (i) cast onto C-PCaP scaffolds with a flat surface, (ii) cast onto C-PCaP scaffolds with grooved surface, which were obtained by adding one layer of parallel C-PCaP struts (spacing = 1.4 mm.), (iii) a microfibre composite that was cast onto the C-PCaP bone-mimetic scaffold (un-anchored microfibres), (iv) a microfibre composite that was cast onto the C-PCaP bone-mimetic scaffold (anchored microfibres). The latter were obtained with the combined MEW and ceramic extrusion printing approach. As additional control, a cylinder made of only GelMA was also tested, to analyse the mechanical strength under shear of a monolithic hydrogel. For each experimental group n = 3–9 samples were analysed.

### 2.10 Scanning electron microscopy (SEM) imaging of the engineered ceramic-hydrogel interface

The morphology of the interface between the microfibre meshes and the C-PCaP, as established in the combined printing approach, was visualised via SEM (Phenom PRO SEM, Thermo Fisher Scientific; accelerating voltage of 10 kV). All structures were kept at –80 °C overnight and freeze-dried to remove water from the cartilaginous compartment, and all samples were cut in half in liquid nitrogen, in order to visualize the longitudinal cross-section of the composite scaffold.

### 2.11 Mechanical characterization of reinforced GelMA (cartilaginous compartment) of osteochondral construct

The compressive properties of the microfibre-reinforced GelMA linked to the C-PCaP scaffold, were measured in unconfined uniaxial compression. A 0.1 N min^−1^ ramp force was applied with a DMA device with mounted compression clamps, until reaching a 50% deformation of the disk-shaped hydrogel-microfibre composite compartment (height 1mm diameter 6mm). Experimental groups consisted of GelMA: (i) pristine, (ii) a microfibre compo-site, (iii) cast onto a C-PCaP bone-mimetic scaffold, (iv) a microfibre composite cast onto a C-PCaP bonemimetic scaffold (un-anchored microfibres), (v) a microfibre composite cast onto a C-PCaP bonemimetic scaffold (anchored microfibres). For each group, n = 5–10 structures were tested. The compressive modulus was derived from curve fitting between 12%–17% strain rate.

### 2.12 Cartilage deposition *in vitro* in the engineered osteochondral plug

#### 2.12.1. Engineered osteochondral plug preparation and culture

In this part, osteochondral scaffolds consisted of a cell-free bone and an ACPC-laden cartilage compartment. The bone-mimetic region was composed of a porous C-PCaP structure, (designed strand-to-strand distance = 0.7mm, diameter = 6.3mm, height of C-PCaP = 3mm), capped with a non-macroporous layer of C-PCaP struts, with an anchored microfibre mesh, prepared as described previously via combined printing. For the cartilage region, a 10% w/v GelMA hydrogel precursor solution in PBS was loaded with 2 · 10^7^ ACPCs ml^−1^, and infused in the reinforcing microfibres linked to the C-PCaP structure. Cells were encapsulated at passage 4. To permit rapid cross-linking, the precursor solution was supplemented with a previously described visible-light responsive photoinitiator [51, 52], composed of 0.5mM tris (2,2′-bipyridyl) dichloro-ruthenium (II) hexahydrate (Sigma—Aldrich) and 5mM sodium persulfate (Sigma Aldrich), and exposed to a 1300 lumen white light lamp for 8 min. Samples were cultured in a chondrogenic medium, consisting of DMEM (Gibco, Life Technologies), supplemented with 1% v/v ITS + premix (BD biosciences), 0.2mM ASAP (Sigma Aldrich), 0.1 *μ*M dexamethasone (Sigma Aldrich), 1% v/v HEPES, 100 U ml^−1^ penicillin, 100 *μ*g ml^−1^ streptomycin (Gibco, Life Technologies) and 10 ng ml^−1^ of recombinant human transforming growth factor-β1 (TGF-β1, Peprotech). Samples were cultured for 6 weeks and harvested at two time points (day 1 and day 42) for subsequent analysis. Medium was refreshed every two days. Neo-cartilage formation in the cartilage-region of the engineered plugs, compared to the constructs composed of cell-laden reinforced GelMA only, was evaluated via immuno-histochemistry and biochemical analysis. The effect of the neo-synthesized matrix over the culture time on the mechanical strength of the interface between the bone and cartilage compartment was also assessed.

#### 2.12.2 Biochemical and histological evaluation of neo-cartilage formation

For biochemical evaluation, samples at week 1 (n = 3–6) and 6 (n = 5–14) of culture were harvested, and the chondral compartment was removed and with a razor blade and digested in papain (Papain from papaya latex, Sigma Aldrich) at 60 °C overnight. Sulphated glycosaminoglycan and DNA contents of the constructs were quantified performing a dimethylmethylene blue (DMMB, Sigma-Aldrich, The Netherlands) colorimetric assay and with a Quan-iT-Picogreen-dsDNA-kit assay (Molecular Probes, Invitrogen, Carlsbad, USA). For histological analysis, samples at day 42 (n = 3) were fixated in 4% buffered formalin. For paraffin embedding, samples were decalcified with 0.5M EDTA disodium salt for 1 day. Dehydration was performed through a graded ethanol series, followed by clearing in xylene, embedding in paraffin, and slicing into 5 *μ*m thin sections with a microtome. Sections were stained with safranin-O and Fast Green to visualize GAGs and collagens. Immuno-histochemistry was performed to visualize collagen type I (primary antibody EPR7785, 0.0022 mg./ml., Abcam) and Collagen type II (primary antibody Col2AI II-II6313, 0.6 mg./ml., DSHB). Endogenous peroxidases were blocked via incubation with 0.3% v/v hydrogen peroxide. Antigen retrieval was performed with pronase and hyaluronidase for collagen type II and collagen type I, respectively, at 37 °C. Subsequently, sections were blocked with bovine serum albumin (BSA, 5% w/v in PBS) for 1 h at room temperature, and the primary antibody was incubated overnight at 4 °C. IgGs were used as negative controls. Horseradish peroxidase-labelled secondary antibodies were added for 1 h at room temperature, and the staining was developed using 3,3-diaminobenzidine. Nuclei were counterstained with haematoxylin and sections were mounted in DPX (Millipore).

For the osteochondral constructs, in order to visualize structure without removing the PCaP scaffold due to de-calcification steps, one formalin-fixed sample was dehydrated through a graded ethanol series and embedded in a methyl methacrylate (MMA) resin. Sections (300 *μ*m thick) were obtained with a saw microtome (Leica SP 1600). Thereafter, all sections were stained with basic fuchsin to assess scaffold morphology. Histological slides were imaged using a light microscope (Olympus BX51, Olympus Nederland B.V.) equipped with a digital camera (Olympus DP73, Olympus Nederland B.V.).

#### 2.12.3 Interfacial adhesion strength at the engineered osteochondral interface after culture

At day 1 (n = 3) and 42 (n = 9), osteochondral structures were harvested and kept in medium to ensure hydration. To determine the strength of the connection at the interface between the cartilaginous compartment and the PCaP-based bone compartment, the same settings that were performed for cellfree structures was applied.

### 2.13 Statistical analysis

Results were reported as mean ± standard deviation. Statistical analyses were performed using Matlab (R2018a, The MathWorks, Inc.). Two-sample independent t-tests were performed to compare the diameter of strands that were printed from different PCaP formulations (NC-PCaP and C-PCaP), bio-chemical production of ACPCs from different struc-tures (chondral and osteochondral constructs), and interfacial shear stress after cultivation with ACPCs for 1 and 42 days. The Wilcoxon rank sum test was performed to investigate the differences of the mechanical properties of PCaP scaffolds having different porosity and material composition (non-crosslinkable and crosslinkable). One-way ANOVA, with the Bonferroni post hoc test was performed to investigate the mechanical properties of produced osteochondral constructs in terms of interfacial shear stress and compressive modulus of the cartilaginous compartment. Additionally, this method was also applied to compare *in vitro* biological activity of cells with PCaP scaffolds (indirect and direct methods). Statistical significance was considered for p < 0.05.

## 3 Results and discussion

### 3.1 Optimization printing parameters of printable calcium phosphate (PCaP) paste

**Figure 3:**
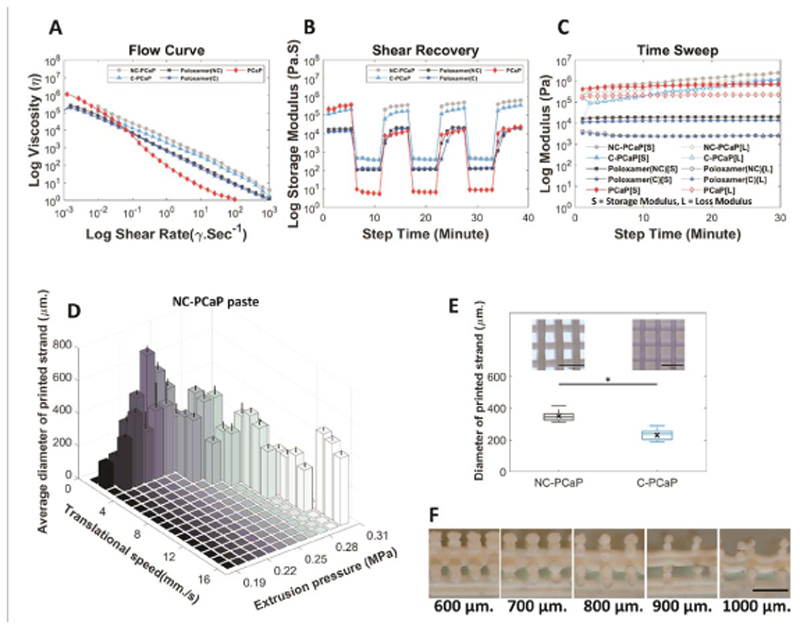
Rheometry and optimization of printing parameters. Rheological analysis highlighting (A) the shear-thinning and (B) shear recovery behaviour of all the inks, and (C) the storage modulus over the printing time, showing no distinctly different behaviour between cements based on the C or NC polymeric carriers. (D) Average diameter of printed strands obtained from two main setting parameters (translational speed and extrusion pressure), (E) comparison between diameter of printed strands fabricated from NC-PCaP paste and C-PCaP paste at the same settings and, (F) the strand-to-strand distance of printable calcium phosphate paste (PCaP). (Scale bar = 1mM.).

First, a ceramic ink was developed to achieve high-resolution patterning and with a setting chemistry compatible with labile polymers and biological compounds. To reach this objective, *α*-TPC was selected as a main material, due to its mild setting reaction [38]. Two formulations of PCaP that could be hardened at physiological temperature were evaluated: one containing a non-crosslinkable poloxamer component (NC-PCaP) and one containing a modified, cross-linkable poloxamer component (C-PCaP). The solid particle to liquid (P/L) ratio of both ink formulations ensured the extruded ink retained its shape and could bear weight after placement, allowing for the formation of multilayer constructs without additional support. These were assessed through rheological characterization, to analyze the flow behavior of the inks when shear forces are applied during printing (figure 3 and supplementary figure S1). When applying shear rates from 0.001 to 1000 S^−1^, viscosity decreases over this range of shear rate. This flow profile shows a comparable shear-thinning behavior for both the NC-PCaP and C-PCaP (figure 3(A)). Additionally, both NC-PCaP and C-PCaP could rapidly recover from applied shears, a condition beneficial to produce high shape fidelity prints (figure 3(B)). For printing, to ensure shape fidelity and uniformity of the printed filaments, printing parameters (extrusion pressure, translational speed) for deposition of the paste were established using the NC-PCaP formulation (figure 3(D)). The optimal printing parameters: MPa, 2mm s^−1^ and 250 *μ*m were selected for the pneumatic pressure, translational speed and layer thickness, respectively. With these parameters, the average diameter of the obtained C-PCaP filaments (230.20 ± 31.24 *μ*m) was close to the inner diameter of the used nozzle (250 *μ*m.), indicating a higher printing resolution than was found for NC-PCaP filaments (349.22 ± 33.56 *μ*m) (figure 3(E)).

Besides printing parameters, shape fidelity in the axial direction is also a pre-requisite for the formation of multi-layered constructs; this factor depends also on the ability of an ink not to undergo deformation when overhanging filaments are stacked without sacrificial supporting materials [53]. Maximum designed strand-to-strand distance for overhanging 90-degree filaments on top of each other without sagging was 800 *μ*m (figure 3(F)). Overall, high shape fidelity was achieved post-printing and upon cement setting, with open and interconnected pores, as well exemplified via *μ*CT (supplementary video SV1). Post-printing, the PCaP ink, which was composed of nanohydroxyapatite (N-HAp) and *α*-tricalcium phosphate micro-particles (*α*-TCP), sets into a cement at physiological temperature, thanks to the hydrolytic conversion of *α*-TCP into calcium deficient hydroxyapatite (CDHA) (supplementary figure S2 and supplementary table 2), and by further crosslinking of the methacryloyl groups in the C-PCaP formulation. While this stabilizes the fabricated construct, the *α*-TCP reactivity and setting initiation could influence the rheology and printability of the ink over time (figure 3(C)). This potential risk can be overcome through tight control of the temperature during the printing process.

### 3.2 Mechanical properties of the biomimetic PCaP scaffolds

**Figure 4:**
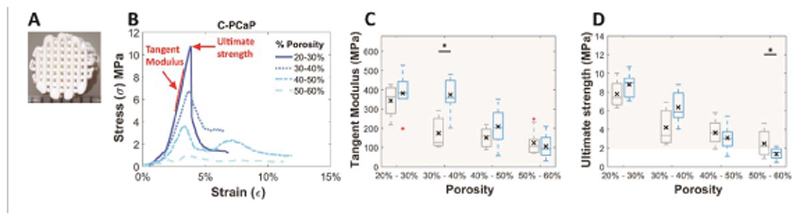
Mechanical properties: (A) representative PCaP scaffold, (B) representative engineered stress-strain curves of C-PCaP scaffolds, (C) tangent modulus, (D) ultimate strength of NC-PCaP paste (grey) and C-PCaP paste (blue) scaffolds with different porosities. (Greyish-filled area showing range of tangent modulus of cancellous bone [10–5000 MPa [54]] (B) and ultimate strength [2–45 MPa [55]] (C)).

After obtaining optimal parameters for printing, mechanical properties of the printed structure (figure 4(A)) are crucial especially for using it as a bone replacement. First of all, the presence of nanohydroxyapatite in the bioprintable paste was not found to significantly alter the mechanical properties of the cement after setting (supplementary figure S3). Next, scaffolds with different ranges of porosity were obtained after printing NC-PCaP and C-PCaP bioma-terial inks following hardening and hardening-crosslinking, respectively. Tangent modulus, ultimate strength and energy to failure were characterized by performing unconfined compression tests and calculated from the stress-strain curves (figure 4(B), supple-mentary figures S4(A) and (B)). Importantly, all formulations and pore designs exhibited compressive properties in the range of cancellous bone [54, 55]. Tangent modulus, ultimate strength and energy to failure of scaffolds made from both NC-PCaP and C-PCaP gradually decreased with increasing porosity, as expected (figures 4(B), (D) and supplementary figure S4(C)). Interestingly, there were no obvious differences in the compressive modulus of scaffolds produced from NC-PCaP and C-PCaP inks, with the only exception of the samples displaying 30%–40% designed porosity. It has been mentioned in the literature that mechanical properties of self-setting ceramics are lower than high-temperature sintering ceramics [56]. Nevertheless, the scaffolds from this study still showed values in the physiological range reported for trabecular bone [54, 55]. While sintering may further improve the mechanical strength of the constructs, this would prevent the direct incorporation and anchoring of lowmelting point thermoplastic polymers as presented in this study as a strategy to improve bone-to-soft tissue interfaces. As such, the high ratio selected for this study (70% w/w of particle content), while giving optimal shape fidelity postprinting, may hinder the formation of a densely crosslinked polymer network, hampering an increase in fracture toughness of the constituent ceramics that could come from the hydrogel covalent crosslink. Nevertheless, considering the overall promising compressive properties and the higher printed filament resolution, C-PCaP was used for the remaining part of this study.

### 3.3 *In vitro* evaluation of bioactivity using mesenchymal stromal cells (MSCs)

**Figure 5:**
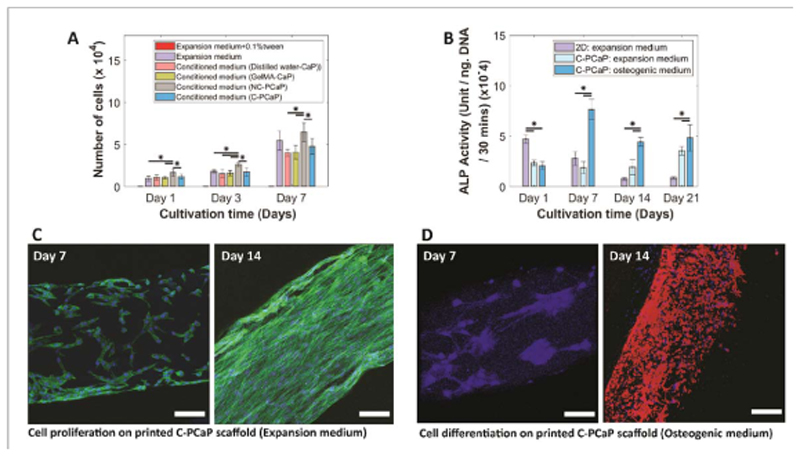
*In vitro* evaluation of bioactivity of the PCaP scaffold. (A) The effect of possible harmful release from composite CaP-based material contained different polymeric carriers on the number of viable cells, (B) the potential of osteogenic differentiation of equine MSCs was investigated through ALP activity, (C) cell proliferation on the C-PCaP filament after cultivation for 7 and 14 days (nucleus (dapi: blue) and F-actin (phalloidin: green)), and (D) cell differentiation on the C-PCaP filament toward an osteogenic lineage after cultivation for 7 and 14 days in an osteogenic medium (nucleus (dapi: blue) and osteonectin protein (osteonectin: red)) (scale bar = 100 *μ*m.).

Cytocompatibility and osteogenic potential of the bone constructs (figure 5) was assessed *in vitro*, using equine bone marrow-derived cells, which were selected in the perspective of future *in vivo* analysis, as the horse is a well-accepted-respected model for evaluating cartilage and osteochondral repair therapies [57–59]. The effects of the release of potentially harmful components was investigated through the culture of MSCs in PCaP conditioned medium, using formulations of the cements that feature different polymeric carriers in the liquid phase. Although free poloxamer above a certain concentration can be harmful [60], our data indicates no negative effect, suggesting no release of detrimental degradation products from the crosslinked poloxamer network or uncontrolled pH changes due to ions released by an incomplete setting reaction of the *α*-TCP microparticles. There was an increase in number of viable cells from day 1 to day 7 in all experimental groups (figure 5(A)) and there were no statistically significant differences after 7 days between the poloxamer-CaP conditioned medium, the positive control (fresh culture medium), and a CaP control with an embedded well-known biocompatible polymer (GelMA). Importantly, MSCs were able to proliferate when seeded directly onto the C-PCaP scaffolds, as indicated by lactate dehydrogenase (LDH) activity (supplementary table 1). Moreover, osteogenic differentiation of equine MSCs cultured on C-PCaP scaffolds was observed after 21 days of culture. The expression of alkaline phosphatase (ALP), an early marker of osteogenic differentiation [61], increased upon MSC culture directly on scaffolds, with higher values and characteristic early peak detection at 7 days in medium supplemented with osteogenic factors (figure 5(B)). Cell proliferation was confirmed via immunofluorescence, observing confluent cell layers on the printed struts that displayed an elongated morphology and developed actin stress filaments after 14 days of culture (figure 5(C)). This is in line with previous studies involving scaffolds using comparable ceramic base components [38]. Importantly, upregulation of osteonectin, a marker of maturing osteoblasts and a hallmark of bone deposition, was detected starting from day 14 in samples with osteogenic medium (figure 5(D), see supplementary figures S4(D), (E)). Overall, the data confirms that the selected PCaP formulation and scaffold have the potential for osteoregeneration, in line with results reported on other bioceramic materials with similar chemical composition.

### 3.4 Fabrication and mechanical properties of the engineered cartilage-bone interface

**Figure 6:**
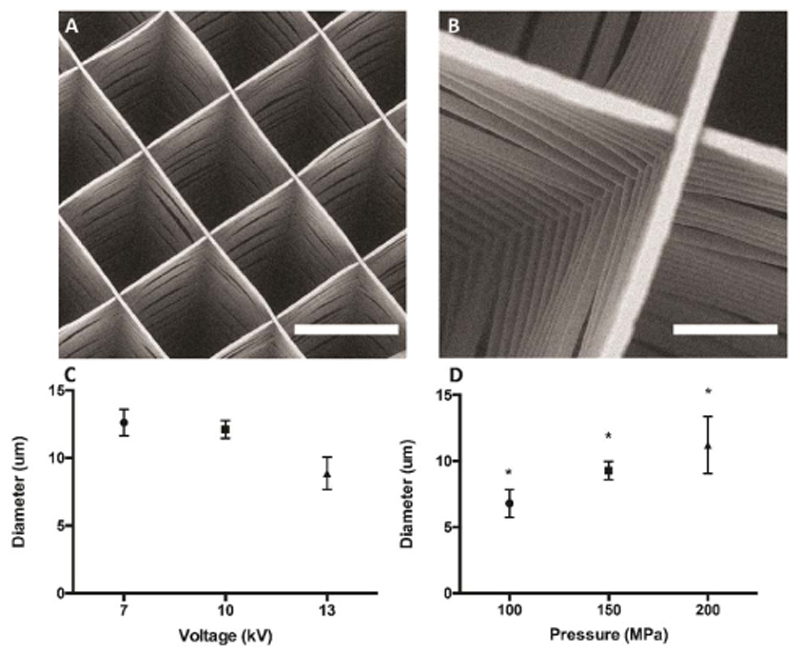
Micro-structure and printing parameters of the MEW fibrous scaffold. (A) and (B) SEM micrographs showing the architecture of the microfibre mesh produced by MEW (A: scale bar = 300 *μ*m., B: scale bar = 50 *μ*m), (C) Relationship between voltage and diameter of PCL microfibre for printing the MEW microfibre mesh. (D) Relationship between pressure and diameter of PCL microfibre for printing MEW microfibre mesh.

For proper integration, it is crucial that the deposition of C-PCaP ink does not alter the organized structure of PCL-microfibre mesh (figure 6). Additionally, preservation of the MEW-printed architecture and microfibre alignment is fundamental to control the mechanical reinforcing effect against compression provided by the PCL mesh when soft hydrogels are embedded in it [62]. Therefore, the initial height for the deposition of the first layer of C-PCaP was set to 80% of total mesh height. Thanks to the fluid paste-like rheological behaviour of the ceramic ink before setting, the material is able to form an interpenetrated structure with the PCL mesh, without altering the microfibre organization and with no detectable effect on the shape fidelity of the extruded ceramic filaments.

**Figure 7:**
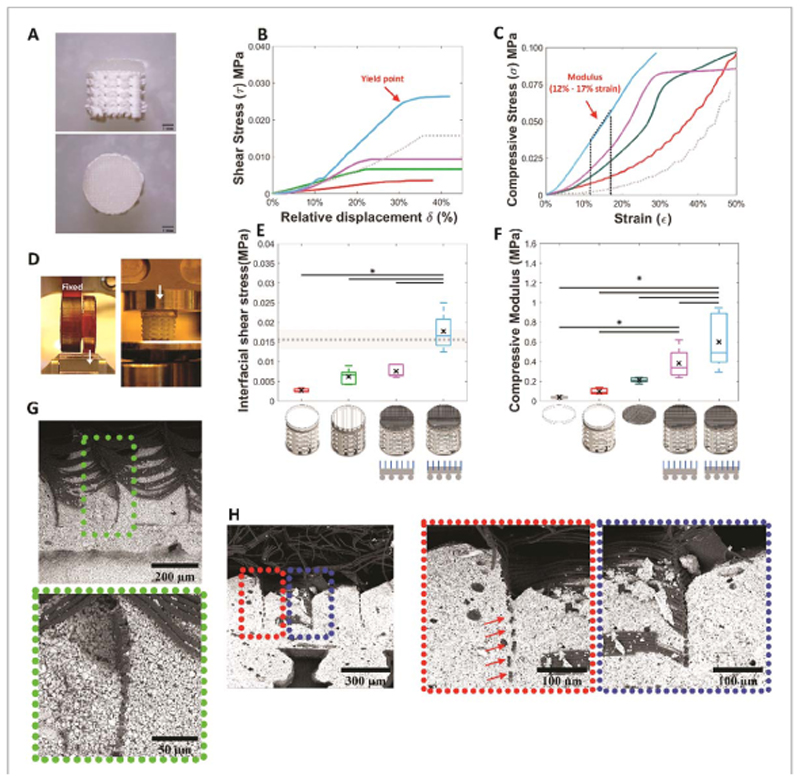
Mechanical properties of the osteochondral unit. (A) Osteochondral unit (scale bar = 1mm.), (B) representative stress-displacement curves from interfacial shear stress assessment at the interface between the chondral and bone compartment, (C) representative stress-strain curves from compression assessment of chondral compartment, (D) mechanical testing (interfacial shear stress: left, and compressive modulus (right)), (E) interfacial shear stress of an engineered osteochondral unit showing alterations due to differences in either interfacial architecture or compositions (GelMA on ceramic (unmodified surface; red), GelMA on ceramic (modified surface; bright green), microfibre reinforced GelMA on ceramic (non-anchor fibre; pink), microfibre reinforced GelMA on ceramic (anchor fibre; blue) and monolithic GelMA hydrogel (mean (grey dotted line) ± SD (grey filled area))), (F) compressive modulus of chondral compartment showing alterations due to difference in composition (GelMA alone (grey), GelMA over fiat interfacial surface of PCaP (red), microfibre reinforced GelMA alone (dark green), microfibre reinforced GelMA on ceramic (non-anchor fibre; pink), microfibre-reinforced GelMA on ceramic (anchor fibre; blue)), (G) SEM micrographs of cross sections of an osteochondral unit revealing embedded microfibres within non-macro porous layer of the bone compartment of newly fabricated structure and (H) after interfacial shear stress assessment.

After the setting of the C-PCaP, the PCL-ceramic ordered composite is formed, with the microfibres anchored into the cement phase and protruding in an ordered fashion into the cartilage region of the osteochondral plug, in which the GelMA hydrogel is lodged by a simple injection (figure 7(A)). The strength of the interconnection (figure 7(B)) at the engineered ceramic-hydrogel interface and the compressive modulus of the chondral compartment (figure 7(C)) were evaluated by using the systems in figure 7(D), and analysing the yield point under interfacial shear stress (figure 7(E)) and the compressive modulus (figure 7(F)), respectively. The interfacial strength of the structures was significantly improved compared to conditions in which the hydrogel was either cast on a smooth or grooved pristine PCaP osteal part, or when the reinforcing microfibres were laid on top of but not anchored into the PCaP (figure 7(E)). The embedding of the MEW reinforcing microfibres within the bioceramic resulted in an approximately 6.5-fold increase, from 2.7 ± 0.5 kPa for the GelMA casted on top of the ceramic, without microfibre interlocked within ceramic, to 17.7 ± 2.0 kPa for the condition in which the fibres were embedded within the ceramic scaffold.

Evaluation of the interfacial toughness showed a similar trend as the interfacial strength (supplementary figure S5). Interestingly, upon mechanical failure of the interface, the microfibres remained well organized and anchored within the bioceramic material, as found by microstructural observation via scanning electron microscopy (SEM) (figures 7(G) and (H)). Collapse upon shear occurred due to loss of adhesion integrity and delamination of the sole hydrogel component.

**Figure 8:**
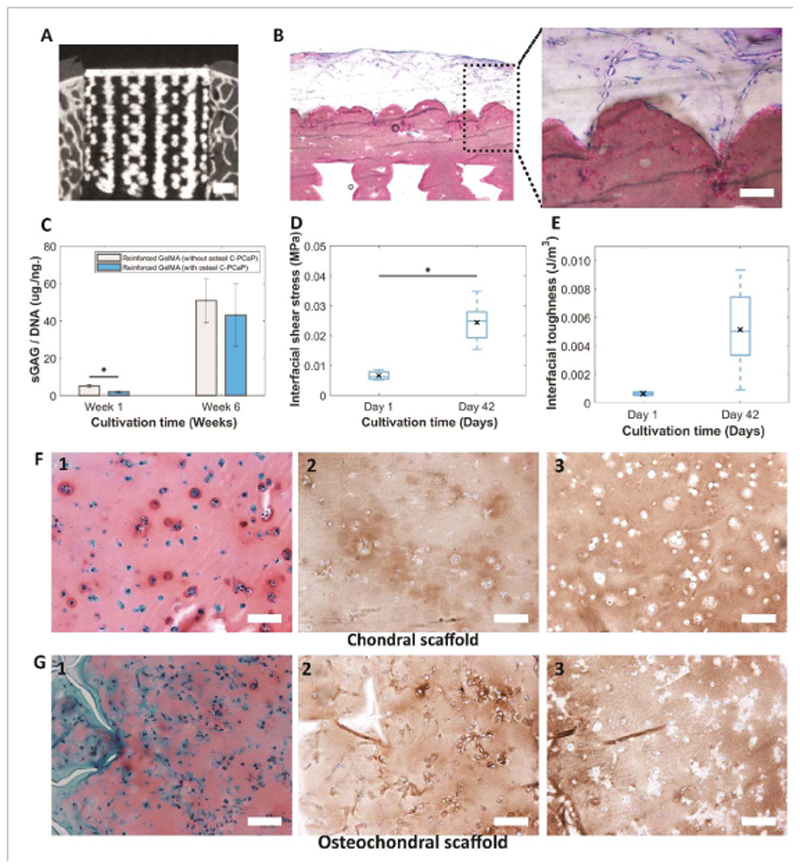
Cartilage deposition in vitro in the engineered osteochondral plug. (A) Micrograph obtained from micro-CT scanning showing a biomimetic PCaP scaffold that could be placed press-fit inside an ex vivo osteochondral defect. (Scale Bar = 1mm.), (B) basic fuchsin and methylene blue staining reveal pattern of embedded PCL microfibres inside the non-porous layer of the C-PCaP scaffold of the constructs with osteal C-PCaP anchor. (Scale Bar = 100 *μ*m.), (C) quantification of sGAG in hydrogel per DNA content. (D) Interfacial adhesion strength and (E) interfacial toughness (day 1 and day 42) while applying shear force at the interface between equine ACPCs encapsulated in GelMA and C-PCaP-based bone compartment. (F1), (G1) Safranin-O staining, (F2), (G2) collagen type II immunostaining and (F3), (G3) collagen type I immunostaining of paraffin embedded microfibre reinforced GelMA without osteal C-PCaP (F) and with osteal C-PCaP (G), respectively, after cultivation for 42 days. (Scale Bar = 100 *μ*m.).

The observed yield shear stresses were slightly above that of GelMA itself (15.6 ± 2.4 kPa), as measured by submitting a monolithic GelMA hydrogel to the shear test. In contrast, for the biphasic hydrogel-bioceramic the fracture was propagated along the interface between hydrogel and bioceramic. Taken together, these results suggest that the MEW microfibrous mesh acts as a bridge between the bony and cartilage compartment in the engineered plug, and that the stability of the interconnection could be further improved employing hydrogels with higher shear strength than GelMA, as well as with strategies to covalently graft the hydrogel component to the thermoplastic microfibres [63]. An important implication of using MEW-microfibres is their ability as reinforcing elements, to remarkably improve the mechanical properties of otherwise soft hydrogels. Previous work demonstrated the ability to enhance the stiffness of GelMA-based constructs, reaching compressive properties mimicking those of native cartilage [31], while computational modelling unravels the mechanisms beyond this behavior [62]. In line, in the present study, an increment in compressive modulus was observed for the microfibre-reinforced GelMA structures (figure 7(F)), with the orthogonal boxes structure architecture selected for the MEW-printed meshes. Importantly, properties were even further improved when the microfibres were embedded within the bioceramic scaffold (3.2-fold versus reinforced hydrogels alone, figure 7(F)), approaching the values of healthy human knee cartilage [64]. This was likely achieved through the stabilisation of the base of the MEW-printed structure and facilitated load transfer to the PCaP scaffold. Such stabilization could prevent early bucking of the stacked layers of microfibres, which has been identified as the main cause of failure of MEW box-shaped meshes under compressive loads [62]. Also, the stabilization of the MEW fibres within the ceramic scaffold allows a more effective lateral confinement of the GelMA hydrogel upon axial compression, thus resulting in a stiffer response. Although interfacial strength is still lower than those found in the native, mature bone-cartilage boundary [65], this mechanical stabilization and reinforcing effect greatly facilitates the surgical handling of the engineered cartilage construct, as well as its implantation *in situ* by press-fitting into an osteochondral defect in a tissue explant model (figure 8(A)).

To further investigate the potential of the multiscale composite osteochondral plugs for the formation of cartilage-like matrix *in vitro*, the chondral reinforcing meshes were infused with articular cartilage derived progenitor cells (ACPC)-containing GelMA and constructs were cultured for 6 weeks. Constructs with (figure 8(B)) and without the osteal C-PCaP anchor were tested, to evaluate the possibility to obtain neo-cartilage in the presence of a bone-supporting material. ACPCs remained viable within the microfibre reinforced GelMA and the deposition of the cartilage-like extracellular matrix was observed in both structures after 6 weeks of culture (figure 8(C)).

Additionally, the neo-synthesized matrix influenced the strength of the interconnection at the bone-cartilage interface of the cell-laden grafts, which improved approximately 3.7-fold from 6.6 ± 1.7 kPa at day 1 to 24.4 ± 6.5 kPa at day 42 (figure 8(D)). Interfacial toughness showed a similar trend (figure 8(E)). Histological evaluation by means of safranin-O staining revealed sGAG deposition (figures 8(F1), (G1)). Collagen type II (Col II) production was also detected in both chondral (figure 8(F2)) and osteochondral constructs (figure 8(G2)), respectively. Collagen type I (Col I) deposition was also detected via histological analysis (figures 8(F3), (G3)). Collagen I is often present as an immature marker in GelMA-based con-structs [37, 66] and can be reduced by incorporation of hyaluronan into the hydrogel matrix [67]. These results underscore that the differentiation of ACPCs towards the chondrogenic lineage is not hampered by the calcium phosphate-based scaffold, suggesting that the construct can be safely used for testing of osteochondral repair techniques.

Overall, a dual reinforcing effect (compression stiffness and interfacial shear strength) was achieved using the combination of ceramic extrusion printing and microfibre electrowriting. Moreover, the coordinated fabrication of such organized, multi-scale composite structures offers new possibilities for functional restoration of damaged osteochondral units. This approach can be further refined by tuning both biological and mechanical properties of the constructs, taking advantage of the physiological setting kinetics of the PCaP ink. Besides facilitating the formation of a tight engineered cartilage-to-subchondral bone connection and supporting osteogenesis *in vitro*, low-temperature setting cements hold the potential to incorporate growth factors (i.e. to enhance osteoinductive and angiogenic properties [68], or even the simultaneous printing of ceramic and hydrogel-embedded living cells [26]). With this in mind, the co-printing in a single biofabrication process of cellfriendly ceramics, cellladen hydrogels and electrowritten microfibres, can be envisioned to comprehensively capture the architecture of native tissue interfaces. In fact, although in this study GelMA was infused in the chondral compartment of the construct, MEW and extrusion-based bioprinting can already be converged in a single biofabrication process, for instance to mimic phenotypic gradients within tissues, such as the zonal cell distribution in articular cartilage [27]. Likewise, as more convoluted microfibre reinforcement geometries can be produced in the hydrogel compartment, specifically designed microfibre motifs could be incorporated to further enhance shear resistance [69], or even to improve tensile behavior [70], the latter with potential application towards the regeneration of tendon and ligament-to-bone interfaces.

## 4 Conclusions

In this study, we demonstrate a novel approach to mechanically integrate hydrogel-based soft tissues to a stiff, bone-like material with potential application for the regeneration hard-to-soft tissue interfaces, in particular in case of osteochondral plugs. To achieve this, a multiscale printing approach, combining ceramic extrusion 3D plotting and the electrowriting of thermoplastic microfibres, was developed. Importantly, the mechanical properties of each compartment (bone, cartilage, interface) can be controlled through the internal architecture of both the reinforcing microfibre mesh and porous bioceramic by means of printing. Additionally, such an approach relying on low-stiffness electrowritten meshes, provides hydrogel strengthening and compressive properties comparable to native cartilage, without shielding cells from beneficial mechanical loads. Owing to the compatibility of the operating physiological temperatures and environmental conditions used for the printing and setting of the PCaP ink, direct anchoring of electrowritten PCL structures in the cement material could be achieved. All materials used, as well as the composite structure, had no impact on cell survival and hence permitted bone and cartilage engineering *in vitro*. This approach offers a promising opportunity for designing interfaces and composite materials with multiple applications in connective tissue regenerative medicine. Overall, these results provide important cues for the biofabrication of a next generation of multimaterial, composite tissues and interfaces, which could integrate 3D printed elements mimicking living tissues down to the micron range.

## Supplementary Material

Figure S1

Figure S2

Figure S3

Figure S4

Figure S5

Supplementary material

Supplementary video
