# Supplementary figures and images for: Combining multi-scale 3D printing technologies to engineer reinforced hydrogel-ceramic interfaces
Source: Biofabrication. Author manuscript; Available in PMC 2020 Oct 16. (PMC7116207; doi:10.1088/1758-5090/ab69d9)

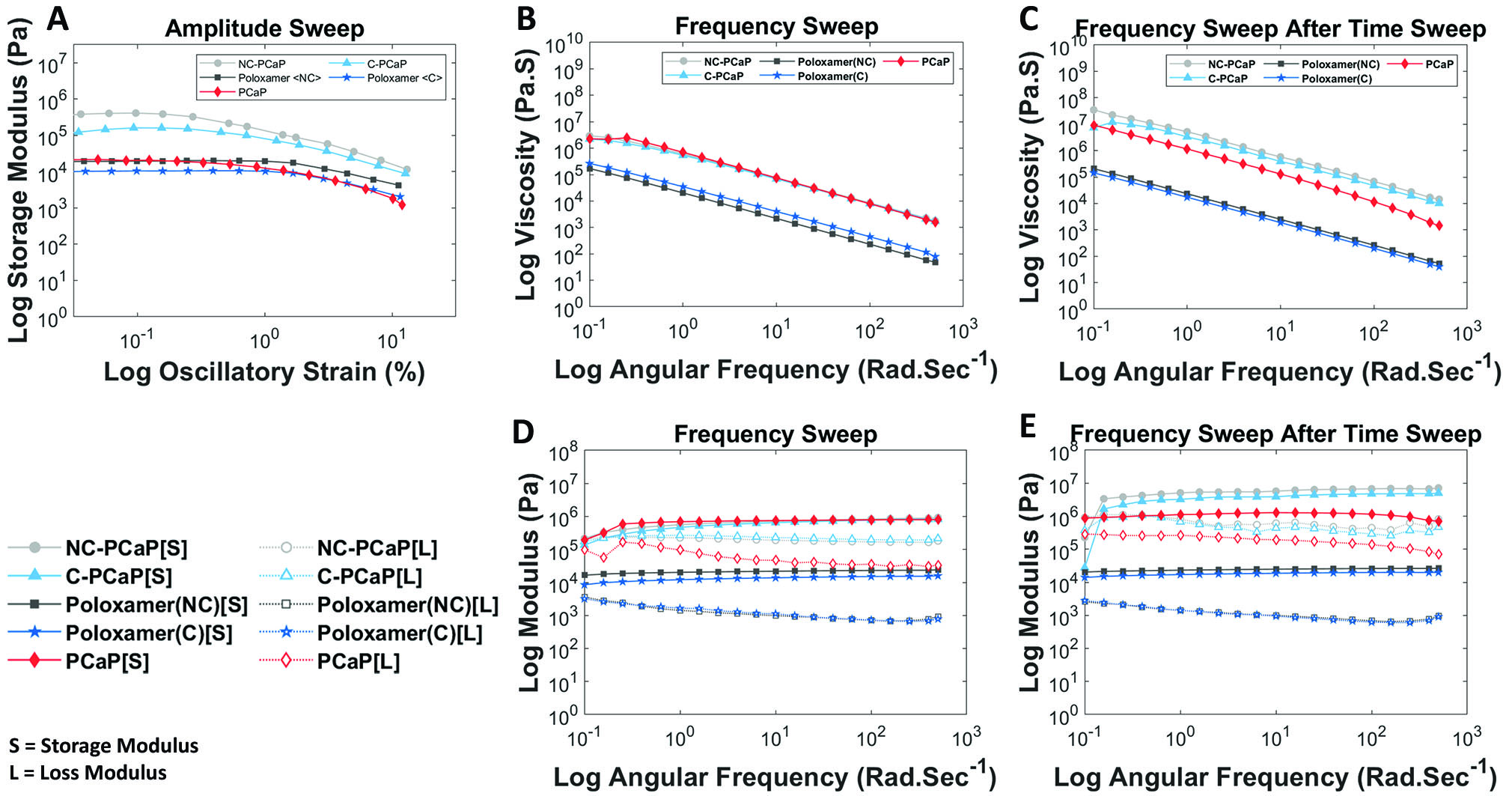

Supplement: Figure S1 [file EMS96600-supplement-Figure_S1.jpg]

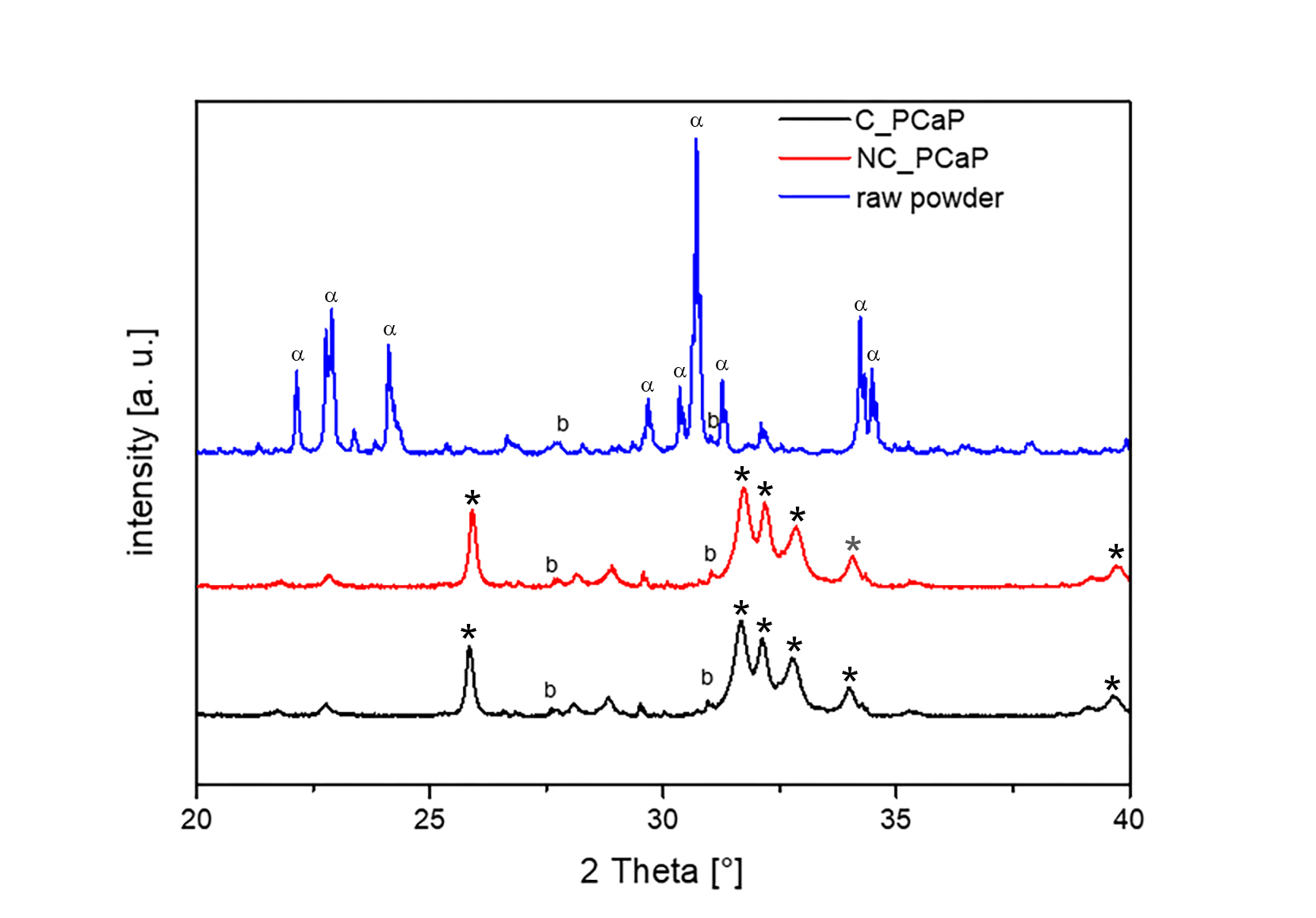

Supplement: Figure S2 [file EMS96600-supplement-Figure_S2.jpg]

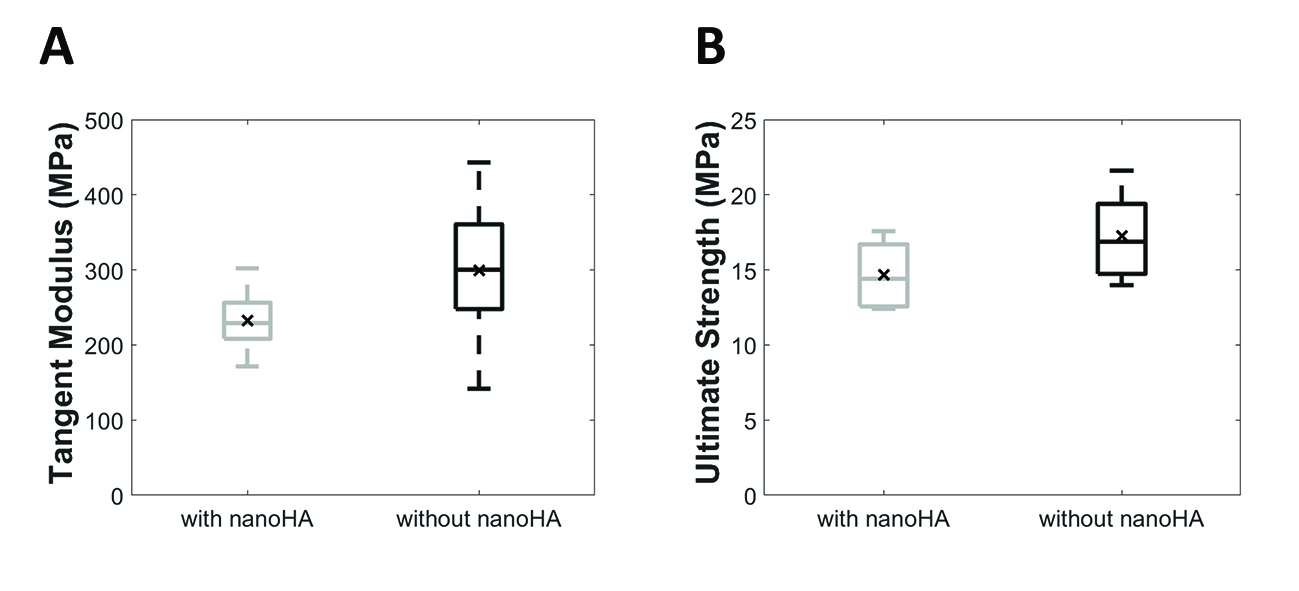

Supplement: Figure S3 [file EMS96600-supplement-Figure_S3.jpg]

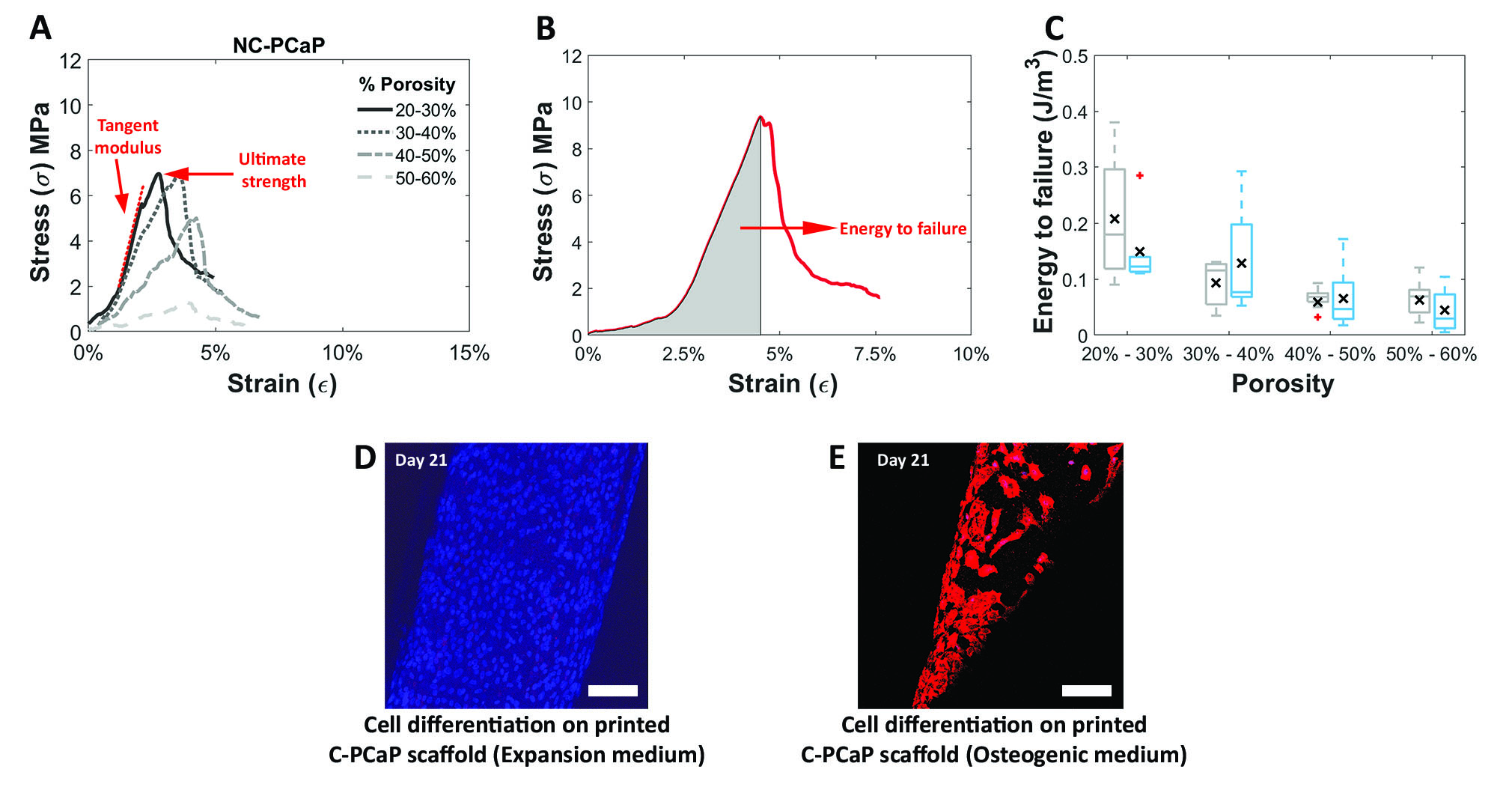

Supplement: Figure S4 [file EMS96600-supplement-Figure_S4.jpg]

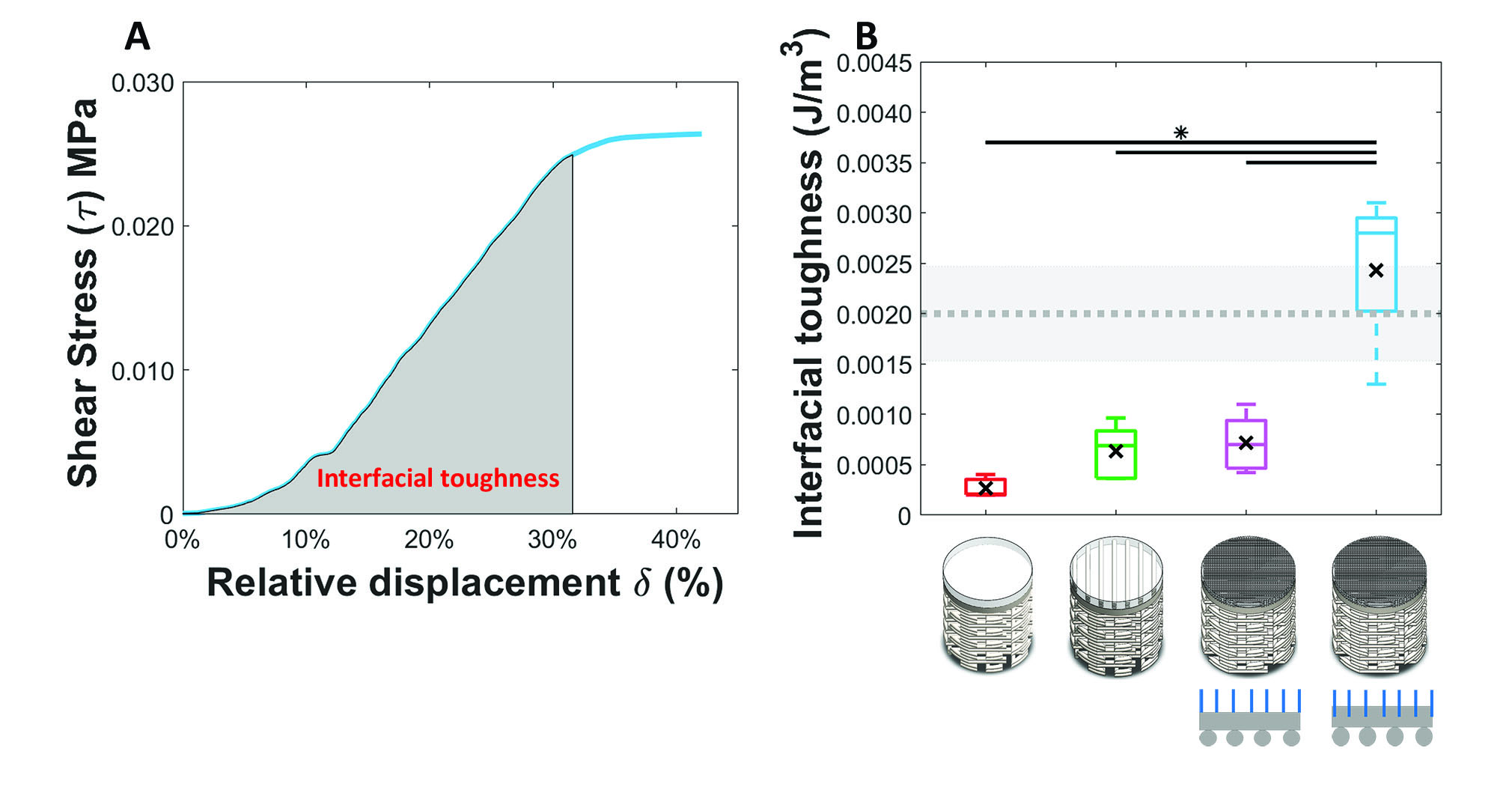

Supplement: Figure S5 [file EMS96600-supplement-Figure_S5.jpg]
