# Supplementary material for: Combining multi-scale 3D printing technologies to engineer reinforced hydrogel-ceramic interfaces
Source: Biofabrication. Author manuscript; Available in PMC 2020 Oct 16. (PMC7116207; doi:10.1088/1758-5090/ab69d9)
Supplement: Supplementary material [file EMS96600-supplement-Supplementary_material.pdf]

Supplementary data

Supplementary methods

1. X-ray diffraction pattern

X-ray diffraction patterns were recorded with a Bruker D8 Advance system (Bruker, Karlsruhe, Germany) in a 2Theta range from 20-40° with Cu Ka radiation (40 KV/ 40 mA) with a step size of 0.02° and a total measurement time of 1 s/step. Quantification was performed by Rietveld refinement analysis using Topas software (Bruker, Germany). The amorphous content of the samples was calculated using the G-factor method with a crystalline corundum reference according to Hurle *et al* [S1].

Supplementary Tables

**Amount of cells**	**Day 3**	**Day 7**	**Day 14**
Mean	1602.62	6568.07	10201.17
Standard Deviation	± 570.00	± 3256.64	± 5992.86

Supplementary Table 1. Proliferation of equine MSCs on C-PCaP scaffolds during cultivation for 14 days

**Sample**	**α-TCP [%]**	**Hydroxyapatite [%]**	**ß-TCP [%]**	**amorph [%]**
Powder	80	1.3	3.7	15
C-PCaP	1.4	62.3	1.4	35
NC-PCaP	1.2	64.7	2	32

Supplementary Table 2: Quantification of the XRD patterns by Rietveld refinement (TOPAS software, Bruker, USA), showing the conversion of α-TCP to an apatite phase.

Supplementary Figures

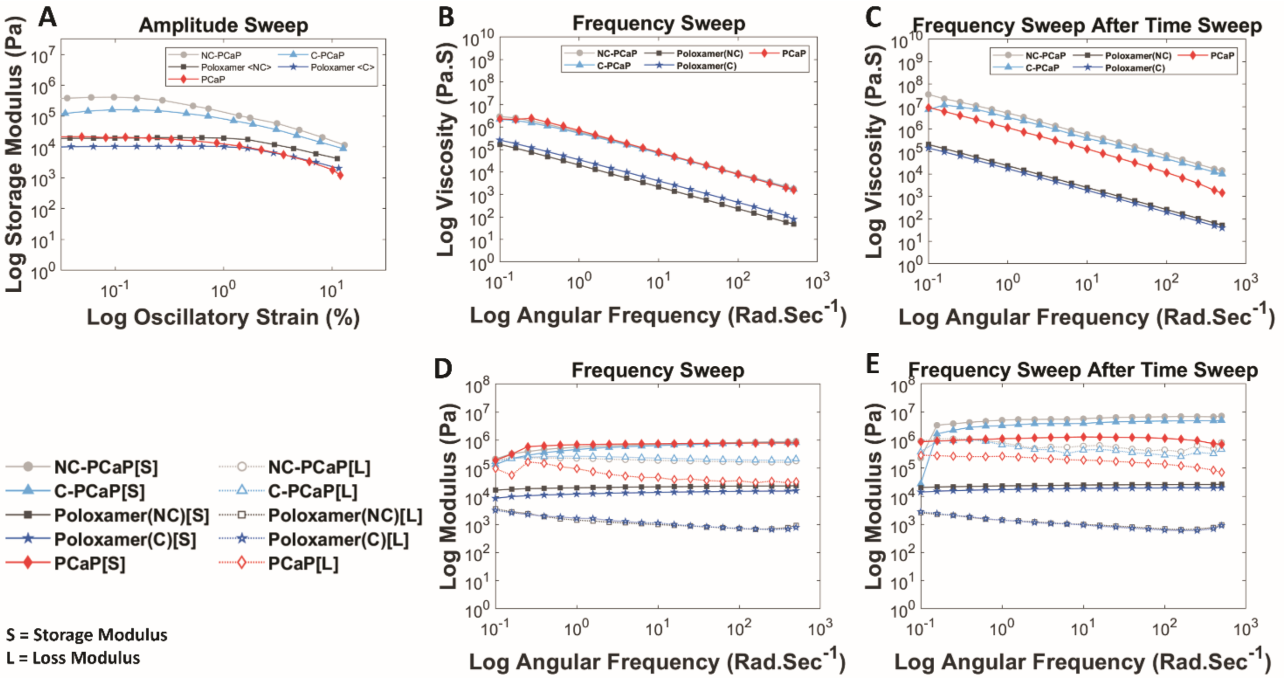

Supplementary Figure S1: A; Amplitude sweep representing LVR, B; Frequency sweep (relationship between angular frequency and complex viscosity), C; Frequency sweep (relationship between angular frequency and complex viscosity, obtained by using same material after performing time sweep test), D ; Frequency sweep (relationship between angular frequency and modulus), E; Frequency sweep (relationship between angular frequency and modulus, obtained by using same material after performing time sweep test).

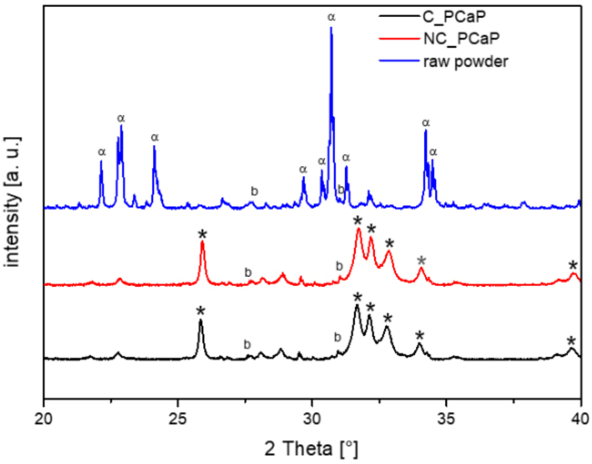

Supplementary Figure S2: X-ray diffraction patterns of raw powder and scaffolds. Diffraction peaks in the raw powder can be assigned to crystalline α-tricalcium phosphate (PDF-No.: 09-0438) with a minor fraction of ß-tricalcium phosphate (PDF-No.: 09-0169, marked with "b"), possibly present as minor impurity in the α-TCP particle formulation. The fabricated scaffolds consisted of low crystalline hydroxyapatite (PDF-No.: 09-0432) from hydrolysis of α-TCP, as shown by the typical broad peaks peculiar of CDHA formation [S2], while the ß-TCP fraction remained unreacted.(α = α-TCP, b = β-TCP, * = CDHA)

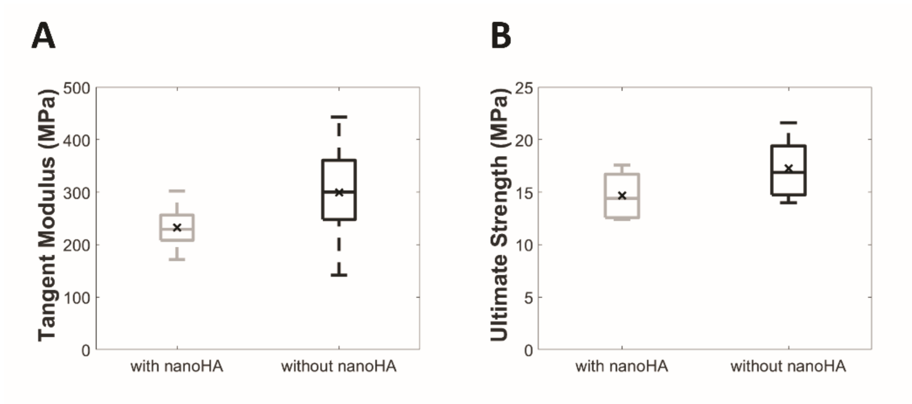

Supplementary Figure S3: Tangent Modulus of hardened cement structure produced from composition of α-TCP with and without nano-hydroxyapatite, B; Ultimate strength of hardened structure produced from composition of α-TCP with and without nano-hydroxyapatite. No significant differences were found between the two groups, suggesting that the added nano-HA does not have a relevant impact on the compressive properties of the produced cement. (n = 6 for each group).

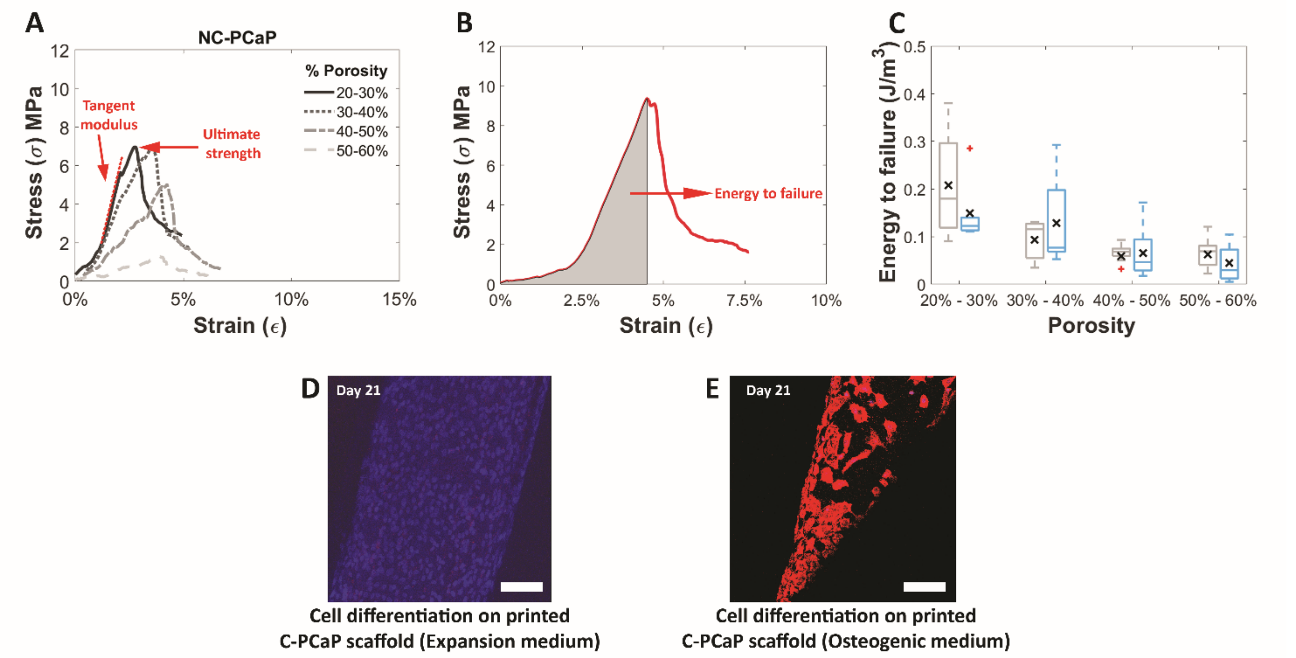

Supplementary Figure S4: A; Representative stress-strain curves of NC-PCaP scaffolds at different porosities showing how to calculate tangent modulus and ultimate strength. B; Representative stress-strain curve showing how to calculate energy to failure. C; Energy to failure of NC-PCaP paste (grey) and C-PCaP paste (blue) scaffolds with different porosities. D; Merged image between fluorescence staining of nucleus (dapi: blue) and osteonectin protein (osteonectin: red) of equine MSCs that were cultured on a C-PCaP scaffold for 21 days in an expansion medium showed no sign of osteogenic upregulation. (Scale Bar = 100 µm.) E; Merged image between fluorescence staining of nucleus (dapi: blue) and osteonectin protein (osteonectin: red) of equine MSCs that were cultured on a C-PCaP scaffold for 21 days in an osteogenic supplement medium showed signs of osteogenic upregulation. (Scale Bar = 100 µm.)

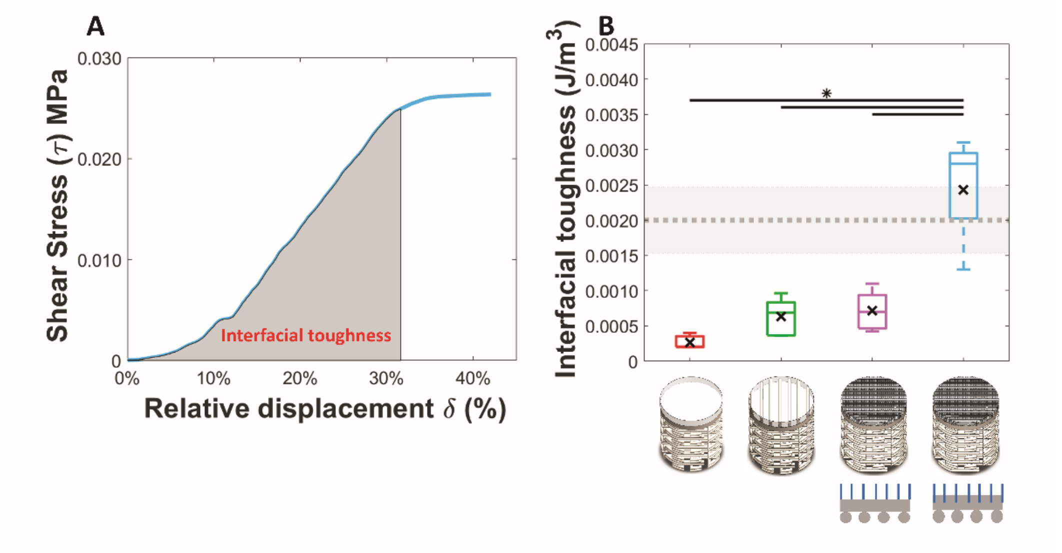

Supplementary Figure S5: A; Representative area under stress-displacement curve for calculation energy to failure. B; Interfacial toughness at the interface between chondral and bony compartment of an engineered osteochondral unit showing alterations due to differences in either interfacial architecture or compositions. The different construct types: GelMA on ceramic(unmodified surface; red), GelMA on ceramic (modified surface; bright green), microfibre reinforced GelMA on ceramic(non-anchor fibre; pink), microfibre reinforced GelMA on ceramic (anchor fibre; blue) and only GelMA hydrogel (mean (grey dotted line) ± SD (grey filled area))

Supplementary Video SV1. Video showing the open and interconnected porosity within the inner structure of the porous 3D printed scaffolds, as shown through a series of μCT sections of the constructs.

Supplementary references

[S1] Hurle, K.; Neubauer, J.; Bohner, M.; Doebelin, N.; Goetz-Neunhoeffer, F, Effect of amorphous phases during the hydraulic conversion of alpha-TCP into calcium-deficient hydroxyapatite, Acta Biomaterialia, 2014, 10, 3931-3941

[S2] Barba A, Diez-Escudero A, Maazouz Y, Rappe K, Espanol M, Montufar EB, Bonany M, Sadowska JM, Guillem-Marti J, Öhman-Mägi C, Persson C, Manzanares MC, Franch J, Ginebra MP. Osteoinduction by Foamed and 3D-Printed Calcium Phosphate Scaffolds: Effect of Nanostructure and Pore Architecture. ACS Appl Mater Interfaces. 9(48) (2017) 41722-36.
